# Total Antioxidant Capacity and Malondialdehyde in Depressive Rotational Shift Workers

**DOI:** 10.1155/2013/150693

**Published:** 2013-04-14

**Authors:** Farahnaz Khajehnasiri, Seyed Bagher Mortazavi, Abdolamir Allameh, Shahin Akhondzadeh, Hassan Hashemi

**Affiliations:** ^1^Department of Occupational & environmental Health, Faculty of Medical Sciences, Tarbiat Modares University, North Kargar Street, Tehran 14117-13116, Iran; ^2^Department of Biochemistry, Faculty of Medical Sciences, Tarbiat Modares University, Tehran 14117-13116, Iran; ^3^Psychiatric Research Center, Roozbeh Hospital, Tehran University of Medical Sciences, Tehran, Iran; ^4^Environment Research Center, Isfahan University of Medical Sciences, Isfahan, Iran

## Abstract

Shift work is associated with sleep deprivation, occupational stress, and increased risk of depression. Depressed patients show increased oxidative stress. During excessive oxidative stress, Malondialdehyde (MDA) increases and total antioxidant capacity (TAC) decreases in body. This cross-sectional study was conducted to determine the serum level of TAC and MDA among depressed rotational shift workers in Shahid Tondooyan Tehran Oil Refinery. 21-item Beck Depression Inventory was used to measure depression level. The level of TAC and MDA was measured by 8 mL fasting blood sample. MDA was determined by thiobarbituric acid reaction. Serum total antioxidants were measured using the ABTS. Results of this study showed that TAC mean and standard deviation concentration was 2.451 (±0.536) mg/dL and MDA was 3.725 (±1.098) mic*·*mol/L, and mean and standard deviation of depression score and BMI were 14.07 (±3.84) and 24.92 (±3.65) kg/m^2^, respectively. Depression score had a positive correlation with rotational shift work experience and work experience (*r* = 0.218 and *r* = 0.212), respectively, (*P* < 0.05).

## 1. Introduction

Shift work is defined as work scheduled outside the normal daytime working hours (7 AM to 6 PM) [1]. The trend in our society is toward an increasing pattern of shift work and it is essential for many industries such as refineries to have 24-hour work pattern [2]. More than 20 to 30 percent of workers are shift workers [3]. They are forced to work and sleep against normal chronobiological rhythms, and as a result the pattern of sleep-wake becomes misaligned [4]. Shift work can alter human Circadian system which is normally synchronized with the solar day [5]. Shift workers can never be adapted to their sleep/activity cycle, which is necessary for their work shift. They sleep at times their organism is set to activity and they work when physical effectiveness is low [6].

Disruption of normal circadian system can cause physiological and psychological problems. Shift work also negatively impacts workers' health condition [7] and cause diseases resulting in absenteeism from work. Sleep disorders are among the health problems caused by shift work [8]. The prevalence of difficulty initiating sleep is higher in rotational shift workers compared with regular day workers [9]. Based on different studies, shift workers complaining about sleep disorders and insufficient sleep range from 10% to 90% [10–12]. Furthermore, sleep disorders and occupational stress lead to more sleepiness and reduce neurobehavioral function consequently, and, therefore, increase the risk of depression [13].

There is an association between poor sleep and symptoms of major depression in male shift workers [14]. Depression and sleep deprivation are reported to cause oxidative stress [15], resulting in the formation of reactive oxygen species (ROS) and eventually lead to neuronal and cellular damage. ROS are formed in the human body in the cytosol, mitochondria, lysosomes, peroxisomes, and plasma membranes under both physiological and pathological conditions [16]; their levels can increase by stress situations such as occupational stress [17, 18]. Stressful conditions lead to the excessive formation of ROS and cause oxidative stress [19]. Oxidative stresses occur when the production of free radicals exceeds the defensive response of the antioxidant system. Oxidative stress has a major role in the causality of some disorders that have higher prevalence in shift workers [20]. Malondialdehyde (MDA) increases in body during excessive oxidative stress [21]. 

Lipid peroxidation is one of the major outcomes of free-radical-mediated injury that directly damages membranes and generates a number of secondary products including aldehydes such as MDA, which is the most abundant individual aldehyde, resulting from lipid peroxidation [22]; also in oxidative stress, total antioxidant capacity (TAC) decreases [23]. Free radicals initiate a cascade, causing lipid peroxidation, DNA damage, cell death, and neurological problems. Total plasma antioxidant capacity is measured as an indicator of oxidative stress [24].

In Iran, few studies have been carried out about shift workers [25, 26]. Most of these studies are concerned with shift workers in Iranian hospitals. Studies about the shift workers in Iranian industries are rare [27]. The results of these few studies have revealed that Iranian shift workers are at risk of depression [28]; so this study was conducted to determine serum level of TAC and MDA among depressed rotational shift workers in Shahid Tongouyan Tehran Oil Refinery.

## 2. Methods

### 2.1. Research Participants

In this cross-sectional study, 456 potentially eligible candidates (all the shift workers in Tehran Shahid Tondgooyan Oil Refinery) were screened for depression symptoms by using 21-item Beck Depression Inventory questionnaire; a total of 397 (87.06%) workers returned the questionnaire. Out of 397 workers, 261 workers did not meet the inclusion criteria or met exclusion criteria**. **Finally, 136 shift workers aged 21–52 enrolled in the study. It should be noted that all of the shift workers in Tehran Shahid Tondgooyan Oil Refinery were men and their program was 8-hour backward shifts (from night to morning) 4 nights, 3 off, 4 afternoons, 1 off, and 4 mornings, respectively.

#### 2.1.1. Inclusion and Exclusion Criteria

The study inclusion criteria were giving written consent to participate in the study and depression score ≥10 in 21-item Beck Depression Rating Scale and washout periods of two months for antidepressants medicines and two weeks for supplements were required, prior to the study entry. The exclusion criteria were history of thyroid diseases, liver diseases, kidney diseases, diabetes, cardiovascular diseases, cancer, and hypertension based physical examination, being professional sportsman, smoking, consuming alcohol, and substance abuse Information was collected using a self-administered general questionnaire.

### 2.2. Data Collection Tools

Data collection was performed using a self-administered general questionnaire “21-item Beck Depression Inventory” [29], which was translated into Persian. The general questionnaire elicited information on age, marital status, work experience, shift work experience, education, sports, smoking, alcoholic drinks, narcotics, and drugs. For enrolled participants, weight and height were measured with Seca standard tools (Germany) with 0.1 cm and 100 g precision while the participants had least clothes and were barefoot and body mass index (BMI) was calculated from the formula (weight (kg)/high (m)^2^).

Diastolic and systolic blood pressures were measured using a mercury sphygmomanometer with 5 mmHg precision from the right arm and after 10 min of resting in the sitting position. History of thyroid diseases, liver diseases, kidney diseases, diabetes, cardiovascular diseases, cancer, and hypertension was collected based on physical examination performed by the interviewing physician in the Health Center of Tehran Shahid Tondgooyan Oil Refinery.

#### 2.2.1. Blood Sample Collection

To measure the level of TAC and MDA, 8 mL blood sample was collected from the vein anterior to the elbow at sitting position and after fasting for 10–12 hours from 7.30 AM to 8.30 AM o'clock. Needle holder 21 in a gel-containing tube without anticoagulant was used. Then, serum was separated using centrifuge for about 10 min at 1500 rpm. The extracted serum was transferred to microtubes labeled with identifier code and was kept at −70°C until the analysis time. The serum MDA level was determined using the method described by Satoh [30]. In this method, MDA was determined by thiobarbituric acid (TBA) reaction and separation on HPLC. UV detection was performed at 532 nm.

Serum total antioxidants was measured using the ABTS (2,2-Azino-bis sulfonic acid). This method is based upon reconstruct cation ABTS (the maximum absorbance at wavelengths 820, 734, and 660 nm) and the chain-breaking antioxidants are of low molecular mass. When the radical cation ABTS (green-blue) is restored to become a colorless solution, it reduces the optical absorption, proportional to the total antioxidant capacity of serum or plasma [31].

For the blinding purpose, we used samples with the identifier code. TAC was measured in the Department of Biochemistry, Tehran University of Medical Sciences, and MDA was measured in Tehran Noor Research Center. 

### 2.3. Statistical Analyses

All analyses were performed using Statistical Package for Social Sciences (SPSS) version 16.0 for Windows (IBM Corporation, NY, USA). Descriptive statistics were shown as mean and standard deviation. The relationship between quantitative variables was tested by bivariate analysis. Linear regression was used to demonstrate the relationship of depression score with shift work experience. Difference between groups was tested by one-way regression analyses (ANOVA). The significant level was set at *P* < 0.05.

### 2.4. Ethical Consideration

The Review Board of Tarbiat Modares University approved the study. Ethical approval was obtained from the Medical Ethics Committee of Tarbiat Modares University in Tehran, Iran. All participants gave written consent to participate in the study. Participants were explained that the data are considered as confidential and their identity will not be revealed and the data will not be used except for the research purpose.

## 3. Results

Table 1 shows the demographic characteristics and clinical examination information of participants. All the participants were males, age range from 21 to 52 years. The mean age of the participants was 30.75 year (±7.19 yr) and maximum participants were in the age group of less than 30 years old (62.5%). The educational level of the maximum number of participants was high school diploma (58.8%). Regarding marital status, 64% of participants were married. 80.9% of participants were operational workers and 19.1% were firefighters. The work experience of 61% of participants was less than 6 years; 23.5% had been employed between 6 and 15 years, and 15.4% were employed for more than 15 years. Shift work experience in 67.6% was less than 6 years, 19.1% between 6 and 15 years and for 13.2% between 16 to 25 years.

Depression score ranged from 0 to 29. Mean depression score was 14.07 (±3.84). 90.4% were categorized as having mild depression (depression score between 10 and 18), and 6.9%  (*n* = 13) were categorized as having average depression (depression score between 19 and 29). The participants' BMI ranged from 15.9 to 34.3; the mean BMI of the participants was 24.92 kg/m^2^ (±3.65 kg/m^2^). The mean of the measured markers in serum included mean of TAC being 2.41 (±0.54) mg/dL; mean of MDA being 3.72 (±1.10) *µ*mol/L.

Table 2 shows the association between BMI, TAC, and MDA concentrations with shift work experience. This study determined that, the amount of serum TAC increased by increasing shift work experience and differences in serum levels of TAC between the groups of shift work experience were significant (*P* < 0.05)  (ANOVA).

Serum MDA levels and BMI values in groups with a shift experience 5 years and less, 6 to 15, and 16 years and more, there was no significant difference (all *P* > 0.05) (ANOVA).

**Figure 1:**
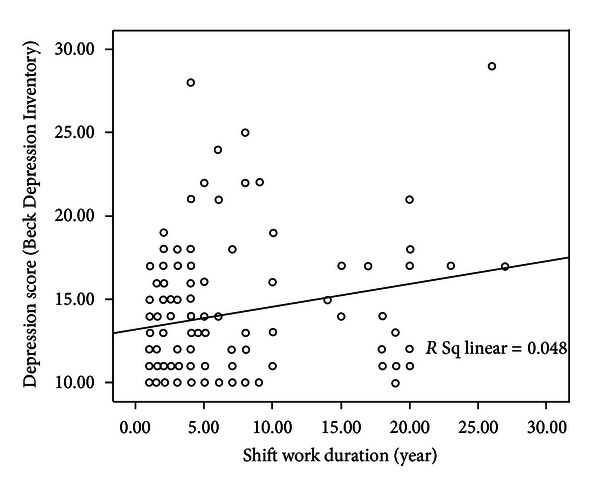
Regression line between depression score and shift work experience.

Figure 1 shows correlation between depression score and shift work experience.

## 4. Discussion

The present study was the first study in Iran (based on the authors' knowledge) that examined the depression in shift workers and its association with oxidative stress markers (TAC and MDA as indicators of oxidative stress). The study was conducted in one of the most important industries in Iran, the refineries. In the present cross-sectional study, the association between age, BMI, shift work experience, marital status on depression score, and total plasma TAC and MDA was assessed.

This study showed that the depression score was more among higher educational level group; this finding was in accordance with a study by Lin et al. which showed that the frequency of major depressive disorder (MDD) was higher among people with higher education [32]. In the present study, depression score had a positive relation with shift work experience (*r* = 0.218, *P* < 0.05). This result is in line with the result of the previous study by Scott et al. [33]. 

Possibly, higher education level of these people has raised their expectation from workplace and absence of expected facilities had caused repulsion and depression among them.

The results of this study did not show any correlation between BMI and TAC, MDA and depression score. While some studies showed that TAC is inversely related to weight [24] and MDA concentration was higher in the obese patients [34], also in some studies BMI and depression were reported to be associated [35] and there is a weak inverse linear trend between obesity and depressive symptoms among men [36]; but some other showed that the association between BMI and depression is nonlinear but U shaped for both genders [37].

Possibly the few numbers in high BMI group have caused this relation not to be seen in this research.

This research did not show any correlation between age and TAC or MDA; however, the study of Sharifian et al. [24] found a correlation between age and total plasma antioxidant capacity through a borderline Pearson's correlation; however, Sharifian's study, the confounding effect of BMI was not controlled and maybe this borderline correlation was related to the impact of BMI. In this study, the mean MDA concentration was 3.72 (±1.10) *µ*mol/L, which is more than 5 times higher than the normal value for MDA, which is less than 0.7 *µ*mol/L [16, 17]. This finding shows that the oxidative stress among these shift workers is much higher than general population. This finding is consistent with the findings of some studies that reported major depression to be associated with increased levels of serum MDA [38, 39].

In summary, the findings showed that mean MDA serum level was higher than reference range and there was a linear relationship between depression score and shift work experience among rotational shift workers, which show a high level of stress and depression among shift workers.

## Figures and Tables

**Table 1:** Relationships between demographic and clinical variable and depression score.

Sociodemographic and clinical variables	Baseline (depression score)	
	Mean (SD)	*P* value^a^
Age (year)	30.75 (7.19)	0.055^a^
Work experience (year)	6.91 (6.76)	0.013^a^
Shift work experience (year)	6.14 (6.24)	0.011^a^
Marital status		
Single (*n* = 49)	13.42 (3.45)	0.15^b^
Married (*n* = 87)	14.42 (4.01)	
Education		
>high school diploma	17.67 (6.66)	
Diploma	13.40 (3.65)	0.024^c∗^
<high school diploma	14.87 (3.78)	
Clinical factors		
BMI kg/m^2^	24.92 (3.65)	0.780^a^
Diastolic blood pressure (mmHg)	66.84 (10.81)	0.111^a^
Systolic blood pressure (mmHg)	110 (13.19)	0.953^a^

^a^Spearman's correlation coefficient.

^b^ *P* value obtained by *t*-test.

^c^ *P* value obtained from one-way ANOVA.

*There are significant differences between diploma and high school diploma < (*P* = 0.029) (Tukey's test).

**Table 2:** Relationship between biomarker concentration at shift work experience (year). (All values are mean SD).

Parameters	≤5 (*n* = 92)	6–15 (*n* = 26)	≥16 (*n* = 18)	*P* value
TAC, g/dL	2.37 ± 0.57	2.55 ± 0.43	2.71 ± 39	0.025
MDA, *μ*mol/L	3.74 ± 1.11	3.82 ± 1.18	3.50 ± 0.88	0.60
BMI, kg/m^2^	24.46 ± 3.80	26.06 ± 3.34	25.61 ± 2.88	0.09

*P value obtained from one-way ANOVA.
